# Editorial: Addressing tuberculosis infection: an essential step in the fight against tuberculosis

**DOI:** 10.3389/fmed.2024.1428677

**Published:** 2024-06-07

**Authors:** Miguel Santin, Anete Trajman, Delia Goletti, Luis Anibarro

**Affiliations:** ^1^Tuberculosis Unit, Department of Infectious Diseases, Bellvitge University Hospital-IDIBELL, L'Hospitalet de Llobregat, Barcelona, Spain; ^2^Department of Clinical Sciences, School of Medicine, University of Barcelona, L'Hospitalet de Llobregat, Barcelona, Spain; ^3^Centro de Investigación Biomédica en Red Enfermedades Infecciosas (CIBERINFEC), Instituto de Salud Carlos III, Ministerio de Ciencia Innovación y Universidades, Madrid, Spain; ^4^Department of Internal Medicine, Federal University of Rio de Janeiro, Rio de Janeiro, Brazil; ^5^McGill TB International Centre, McGill University, Montreal, QC, Canada; ^6^Rede Brasileira de Pesquisa em Tuberculose, REDE-TB, Rio de Janeiro, Brazil; ^7^Translational Research Unit, Department of Epidemiology, Istituto Nazionale per le Malattie Infettive L. Spallanzani-IRCCS, Rome, Italy; ^8^Unidad de Tuberculosis, Enfermedades Infecciosas, Medicina Interna, Complexo Hospitalario Universitario de Pontevedra, Pontevedra, Spain; ^9^Immunology Study Group, Galicia Sur Health Research Institute (IIS Galicia Sur), SERGAS-UVIGO, Vigo, Spain

**Keywords:** *Mycobacterium tuberculosis*, tuberculosis infection, tuberculosis control, tuberculosis preventive therapy, prevention, End TB Strategy, short rifamycin-based regimens

Tuberculosis (TB) still causes 1.3 million deaths each year, despite treatment has been available for over 65 years. Latently infected people constitute the main reservoir of *Mycobacterium tuberculosis* (Mtb) worldwide. Recognizing this, systematic testing and treatment of TB infection (TBI) is central to the World Health Organization's (WHO) “End TB Strategy” to eliminate TB (1). The United Nations General Assembly (UNGA) convened its second High-Level Meeting (UNGA-HLM) on the fight against TB in September 2023 to review the targets set at the first meeting for 2018–2022 and to develop new targets up to 2027, including coverage of TB preventive therapy (TPT) for 45 million household contacts (HHCs) and people living with HIV (PLHIV). However, the limitations of diagnostic tests for TBI in predicting the development of TB (2), the long duration and toxicity of TPT (3), and the lack of resources hinder this strategy from moving forward.

This Research Topic includes eight articles addressing interventions to improve TBI screening and treatment outcomes (Mendes et al.; Ortiz Laza et al.; Alsdurf et al.), challenges to their implementation (Coleman et al.; Liu et al.), the potential utility of antibodies to Mtb-specific antigens for the classification of Mtb infection states (Ruiz-Tagle et al.; Tran et al.), and the feasibility of new approaches to detect viable bacilli across the spectrum of Mtb infection (Alebouyeh et al.).

Increasing human resources, without reducing those available for active TB care, and expanding the use of shorter rifamycin-containing regimens can contribute to the control and eventual elimination of TB. A multicenter cohort study (Ortiz Laza et al.) in Spain showed a sharp decline in TB incidence over the last two decades accompanying the implementation of a TPT program, with a specialist nurse and an electronic registry. Telemonitoring with educational activities, together with the 3 months of rifampicin plus isoniazid therapy, improved TPT completion from <70% to over 85%. However, resources are needed. In a relatively large time and motion study (Alsdurf et al.), a program to scale up TPT in five countries resulted in an average 9% increase in the amount of time HCWs spent on TPT activities, resulting in an 11% reduction in time spent on active disease management. Programs should therefore increase their workforce by 10% to improve TPT activities. New, shorter, and safer regimens are available, but their implementation is slow and, moreover, they are not enough; health system strengthening and training are needed to move things forward. In Brazil, Mendes et al. 14 years after the publication of the national guidelines recommending TPT for contacts of all ages and for PLHIV, TPT is still not fully implemented. The integration of short-course rifapentine plus isoniazid regimen into the health system has been slow. There is debate over extending the TPT to other at-risk populations. A systematic review (Liu et al.) found that people with diabetes have an increased risk of LTBI. Although WHO does not consider diabetes for TB screening, there is evidence of its involvement as a synergistic risk factor for TB when combined with other immunosuppressive or lifestyle factors (4, 5). The debate about whether people with diabetes should be treated for TBI revolves around balancing the increased risk of TB with the challenges in diagnosis, side effects of treatment, and the need for more evidence to inform guidelines. For now, while PLHIV and HHCs of TB are not reached, scaling up TPT for people with diabetes may not be a priority. A review (Coleman et al.) highlights the importance and cost-benefit of identifying and treating people with latent infection to prevent future cases, together with active case finding, to rapidly eliminate all infectious cases. Indeed, WHO has confirmed the benefits and high return of investment of adding TPT to active case finding in countries with different epidemiologic scenarios (6). The authors also summarize the challenges and call for bold strategies to make progress toward TB elimination.

Biomarkers capable of recognizing the early stages of infection would allow targeting individuals with actual recent infection and putting them on TPT. The humoral immune response profile of TB patients and contacts with TBI could serve as a diagnostic tool to differentiate between long-standing and recently acquired TBI, as previously suggested (7). In this Research Topic, two studies address the performance of antibodies against Mtb-specific antigens to discriminate between different TB states. A prospective study investigated Mtb-specific IgA against various antigens in saliva among household contacts of smear-positive pulmonary TB cases (Ruiz-Tagle et al.). They found that levels of some antigens were higher among TB-infected than in uninfected contacts. However, the discriminatory ability of these IgA antibodies specific for TB infection was very low. In contrast, an exploratory retrospective analysis of serum antibodies in a cohort of active TB patients, infected household contacts, and healthy controls (Tran et al.) reported a high sensitivity and specificity of serological tests for TB state classification. The authors found higher serum IgA antibodies to MPT64 antigen, followed by IgG antibodies to Ag85B and CFP, among TB patients than among infected contacts. The results showed a high discriminative accuracy for the MPT64-IgA-based assay (95% sensitivity and 97% specificity). These preliminary results need to be validated in prospective cohorts to formally establish their accuracy in discriminating active TB from TBI.

Finally, admitting the drawbacks of current diagnostic tests for TBI and properly identifying bacterial viability during silent human TBI are major issues to address. A review (Alebouyeh et al.) evaluated the feasibility of new approaches to detect viable bacilli across the spectrum from infection to disease. The authors recognized the limitations imposed by the extremely low bacterial burden in supposedly infected people. Interesting approaches are under evaluation, such as those detecting Mtb DNA in blood by the phage technology (8) or in peripheral CD34+ cells (9).

While dealing with TBI presents challenges, it is essential to maintain an optimistic outlook. Amidst the complexities of latent TB, we must focus on the remarkable progress that has been made and the promise of what lies ahead. Despite the hurdles, the collective commitment to research, innovation, and collaboration continues to push the boundaries of what we can achieve in the fight against TB.

## Author contributions

MS: Writing – original draft, Writing – review & editing. AT: Writing – original draft, Writing – review & editing. DG: Writing – original draft, Writing – review & editing. LA: Writing – original draft, Writing – review & editing.
